# Automatic detection of anchor points for multiple sequence alignment

**DOI:** 10.1186/1471-2105-11-445

**Published:** 2010-09-02

**Authors:** Florian Pitschi, Claudine Devauchelle, Eduardo Corel

**Affiliations:** 1 Partner Institute for Computational Biology, CAS-MPG, 320 Yue Yang Rd, 200031 Shanghai, China; 2 Laboratoire Statistique et Génome (LSG), CNRS UMR 8071, INRA 1152, Université d'Evry, Tour Evry2, Place des Terrasses, 91034 Evry Cedex, France; 3 Georg-August-Universität, Institut für Mikrobiologie und Genetik, Goldschmidtstraße 1, 37077 Göttingen, Germany

## Abstract

**Background:**

Determining beforehand specific positions to align (*anchor points*) has proved valuable for the accuracy of automated multiple sequence alignment (MSA) software. This feature can be used manually to include biological expertise, or automatically, usually by pairwise similarity searches. *Multiple *local similarities are be expected to be more adequate, as more biologically relevant. However, even good multiple local similarities can prove incompatible with the ordering of an alignment.

**Results:**

We use a recently developed algorithm to detect multiple local similarities, which returns subsets of positions in the sequences sharing similar contexts of appearence. In this paper, we describe first how to get, with the help of this method, subsets of positions that could form partial columns in an alignment. We introduce next a graph-theoretic algorithm to detect (and remove) positions in the partial columns that are inconsistent with a multiple alignment. Partial columns can be used, for the time being, as guide only by a few MSA programs: ClustalW 2.0, DIALIGN 2 and T-Coffee. We perform tests on the effect of introducing these columns on the popular benchmark BAliBASE 3.

**Conclusions:**

We show that the inclusion of our partial alignment columns, as anchor points, improve on the whole the accuracy of the aligner ClustalW on the benchmark BAliBASE 3.

## Background

Multiple sequence alignment (MSA) appears as the initial step of almost all biological sequence analyses. However, MSA is well known to be a difficult problem, both from the algorithmic point of view and with respect to the biological relevance of the output. The *local *alignment is a classical paradigm in sequence analysis [1,2]. The idea of including local alignment information into global alignment tools, like in DIALIGN [3], represented an important step in alignment accuracy, and is also at work in more recent tools like T-Coffee [4], MUSCLE [5] or MAFFT [6]. A latest trend is to include homology information retrieved from existing databases, such as, *e.g*., in DbClustal [7]. For recent reviews on MSA programs see [8-10]. Another way to improve the accuracy of existing MSA is to include user-specified *anchor points*, which are specific positions that should turn out to be aligned in the output [11]. This information can be composed of a small number of expert-based constraints, or can be used to include additional information, such as secondary structure predictions (like in [12]) or other information derived from external resources [7].

Multiple sequence alignment, being NP-hard under any reasonable optimisation scheme, must consistently rely on heuristics. The inclusion of anchor points can result in a dramatic improvement on the relevance of the alignment, if it constrains the search of the local optimum to a region that contains the "true" alignment. The number of MSA programs that currently accept the inclusion of user-specified anchor points is unfortunately limited. To our knowledge, only DIALIGN 2 has such an explicit option [13], while it is also possible to include anchor points in ClustalW 2.0 [14], by using the format developed for the BLAST-based BALLAST [7] tool, and in T-Coffee, by including the anchor points as *library files*.

Anchored alignment is also widely used for whole genome alignment strategies, for which it is almost required, by the sheer size of the input, to start by detecting strong pairwise local similarities for the efficiency of the subsequent algorithm (see *e.g*., [15]). For instance, exact maximal repeated substrings (like multi-MUMs or MEMs [16]) can prove to be sufficiently informative, although more recent methods use spaced seeds (see [17]). In this paper, we follow likewise a combinatorial approach, but our focus concerns however not whole genomes, but sequences that are amenable to traditional multiple alignment methods, such as protein or gene-sized nucleic sequences.

We introduce a method to determine automatically a set of such "anchor points" for multiple alignment software. We base ourselves on a previously introduced algorithm, the *N-local decoding*, introduced by Didier [18] that clusters together positions in the sequences whose contexts of appearance of a given length *N *are similar but exhibit an *a priori *unspecified number of mismatches. More precisely, we use a method called MS4 [19], which selects multiple local similarities resulting from the *N*-local decoding, but for an *adaptive *value of *N*.

However, specifying contradicting anchor points can prove deleterious. Indeed, suggesting or imposing that some positions be aligned while these positions are incompatible with the ordering induced by the sequence can altogether destroy the relevance of the alignment. The simplest kind of incompatibility arises from internal repeats inside sequences. The MS4 method is here tuned to accept only similarities occurring at most once in any sequence. Usually, anchor points are specified as pairs of aligned residues, or possibly, of aligned segments. In order not to confuse the reader, we will call the output of this procedure *partial columns*, because they would look as such in a multiple alignment display.

The core of the present paper consists in a graph-theoretic algorithm to tackle the global issues of consistency with a multiple alignment. To do this, we consider the order-theoretic definition of *consistency *(as used, *e.g*., in DIALIGN [20]). Each sequence is seen as an abstract ordered sequence of positions (from left to right). A collection of subsets of positions can be added to a given multiple alignment under a technical condition which ensures that the elements of different subsets never appear in contradicting orders. This condition is readily encoded in a directed graph, and the consistency problem amounts to getting a directed acyclic graph (DAG) from it. Our algorithm starts by implementing a heuristic solution to the NP-hard problem known as the *minimum feedback arc-set problem*. Once a DAG has been identified, positions that contradict the induced partial order are removed from the corresponding partial column. We call the output of this procedure *consistent partial columns*.

As a validation of the method, we introduced the partial columns, and the consistent partial columns, into the programs accepting anchoring options. We tested the effect of introducing these two types of anchor points on the performance of these MSA tools on the global benchmark BAliBASE 3 [21]. The results show that the use of either type of partial columns induce improvements of performance for ClustalW 2.0 on BAliBASE, which are better and stabler with the consistent ones. By contrast, we get a consistent degradation of performance for DIALIGN, and almost no variation for T-Coffee.

Although we used our method only with the MS4-based partial columns, this algorithm can be applied to any other set of partial columns. The MS4 approach has the advantage to detect directly multiple similarities with only linear complexity. Virtually any scheme for detecting local similarities could produce an input for our method, provided that all internal repetitions be removed. It is for example possible to use pairwise similarities, such as used by most MSA programs, and select among them those that involve more than two sequences to construct the partial columns, albeit at some computational cost. In [22], we used the pairwise optimal fragments for DIALIGN, and the consistency algorithm described in the present paper, this time with satisfactory results.

## Methods

Let *S *be a collection of *n *sequences over a finite alphabet. The *site space*

S={(i,p)|1≤i≤n,1≤p≤ℓ(i)},

where ℓ(*i*) is the length of the *i*-th sequence, is the abstract set of positions in the sequences, and is endowed with a natural partial ordering "≼" such that (*i, p*) ≼ (*i'*, *p'*) holds if and only if *i *= *i' *and *p *≤ *p'*. Let *S_i _*be the set of sites of the *i*-th sequence, *i.e*. the set {(*i, p*)|1≤ *p *≤ ℓ(*i*)}. In the following, we identify *S_i _*with the *i*-th sequence.

**Figure 1:**
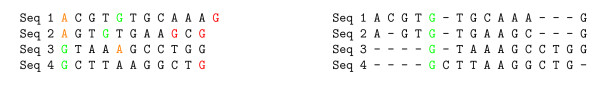
**Alignments as partitions of the set of sites**. (Left) - Unaligned sequences: the class in red is ambiguous. It cannot take place into any alignment of the sequences. The green and orange classes are non-ambiguous. However, they are incompatible with each other. If the green class is accepted, then the orange one must be excluded. (Right) - An alignment of the sequences containing as equivalence class the green subset of sites. Each column of the complete alignment is a subset of sites. The corresponding partition is consistent: in particular, all the elements in a given column appear in the same order with respect to another column.

An *alignment *of *S*, in the sense of DIALIGN [20], is a partition *A *of *S *that satisfies a consistency condition. As usual, we attach to the partition *A *the natural equivalence relation ~*_A _*defined as *x *~ *_A _y *if and only if there exists a subset *a *∈ *A *that contains both *x *and *y*. Then the consistency condition reads as follows: the preorder ≼*_A_*= (≼ U ~*_A_*)*_t_*, where *R_t _*denotes the *transitive closure *of a relation *R*, coincides with the order ≼ when restricted to any sequence *s *∈ *S. *The equivalence classes of the partition *A *correspond to parts of columns of aligned positions of *S*. If only a set of disjoint subsets of positions whose union does not cover the whole set *S *is given, we implicitly consider the partition obtained by adding the missing singletons. These notions are illustrated for concreteness' sake on the toy example presented in Figure 1.

We call a subset C⊂S*ambiguous *if it contains a repetition, that is, there is a sequence $S_{i}$ such that the intersection $C\cap S_{i}$ contains at least two distinct elements (*i, p*) and (*i, p'*), which are then also called *ambiguous *with respect to *C*. This definition is extended to an equivalence relation *E *on S by calling *E *itself *ambiguous*, if it contains an equivalence class which is an ambiguous subset.

A non-ambiguous subset C⊂S will be called a *partial alignment column*. A non-ambiguous equivalence relation consists therefore only of partial alignment columns. If an equivalence relation is consistent, it is obviously non-ambiguous. The converse is however in general not true.

### The MS4 method

Our partial column detection scheme is called MS4, and is described in [19]. It relies on a fast algorithm for producing partitions of sites, the *N-local decoding*, that we briefly recall.

**Figure 2:**
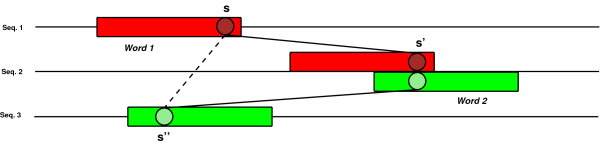
***N*-local decoding**. Schematic explanation of the NLD procedure. Sites *s *and *s' *(resp. *s' *and *s'*') appear at the same position of two occurrences of an identical word (respectively words 1 and 2). Positions *s *and *s'*' are therefore connected by transitive closure.

A word $w\in A^{N}$*occurs at position i relatively to *s = (*s, p*) if *s*_[*p-i, p-i+N*-1] _= *w*. Say *σ *≃ *_N_σ' *whenever there is an identical length *N *word *w *at the *same *position relatively to both *σ *and *σ'*. A single length *N *word induces *N *instances of the relation ≃*_N_*, one for each position in the word. The *N-local decoding of *S is the partition ℰ^*N *^of S induced by the transitive closure of ≃*_N_*. Therefore, two sites *σ *and *σ' *are clustered together if there is a chain of occurrences of identical length *N *words that connects them (Figure 2).

The MS4 method combines the different equivalence classes from various values of *N *by introducing a new construction, the *partition tree*, which encodes how the equivalence classes for successive values of *N *are related.

Letting ${ℰ}^{0}=\{S\}$ we can encode the set *V *= U_*i*≥ _ℰ*^i ^*of equivalence classes for different values of *N *into the *partition tree ***P **= (*V, E***^P^**), defined by

$$E^{P}=\{(u,v)\in {ℰ}^{N}\times {ℰ}^{N+1}|v\subset u\}.$$

The leaves of the partition tree are the sites in the sequences. Let us say that a node is *ambiguous *if the leaves of the partition tree that are children of this node form an ambiguous subset of sites.

**Figure 3:**
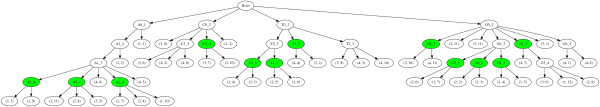
**Selection of partial column subsets on a partition tree**. Toy example (continued) - Selection of nodes corresponding to partial columns using the partition tree. The partition tree of the example in Figure 1 features as green nodes the "highest" *non-ambiguous *nodes. The leaves are the sites (*i*, *p*) in the sequences *i *at position *p *(note that numbering starts with 0). Sets of leaves sitting underneath a green node correspond to selected partial alignment columns. An inner node is denoted by a suffixed letter, *e.g*., A2_-_4 corresponds to an equivalence class for *N *= 4. The green nodes share the property of being non-ambiguous and having an ambiguous node as direct ancestor.

**Figure 4:**
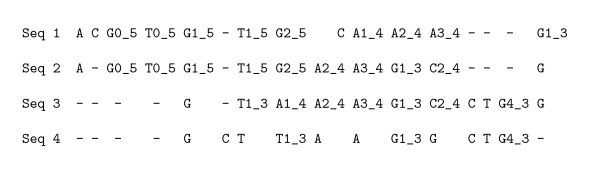
**Alignments as partitions of the set of sites (continued)**. Toy example (continued) - The example alignment of Figure 1, onto which the partial columns detected by MS4 have been "projected". Some MS4 classes, for instance T0_-_5, correspond exactly to some equivalence class of the original alignment, while others, like G2_-_5 form only a subset of an alignment column. Note that other classes, like G1_-_3, while being partial columns by definition, are not part of this particular alignment.

**Figure 5:**
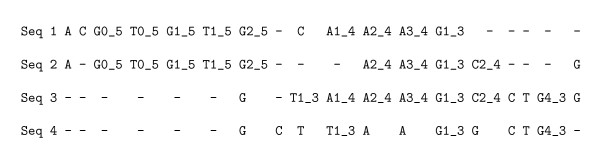
**Alignments as partitions of the set of sites (end)**. Toy example (end) - The alignment of the same sequences as produced by DIALIGN 2 with the featured partial columns included as anchor points. Almost all MS4 partial columns are now part of the alignment, except T1_-_3. The set of MS4 partial columns for this example is consistent, as can be seen by simply sliding the lowest occurrence of T1_-_3 below its corresponding fellow. The perturbation in the alignment is however sufficiently penalized by the optimisation function of DIALIGN to be rejected from the final alignment.

Given k ∈ ℕ, we define the set of *MS4 partial columns *(or shorter, *partial columns*) spanning at least *k *sequences, as the set of subsets of sites $C_{k}$ corresponding to the children of non-ambiguous nodes *v *of the partition tree such that their direct ancestor in the tree is ambiguous (see Figure 3). This condition ensures that the resulting subsets of sites are indeed partial columns, as illustrated in Figures 4 and 5.

### Consistent Partial Columns

We present now the algorithm that resolves the inconsistencies among a set of partial columns.

The *succession graph *of a set C of partial columns is the edge-weighted directed graph SG(C)=(C,E,w) where we have an edge e = (*C, C'*) if and only if there exists a sequence *i *and sites (*i, p*) ∈ *C *and (*i, p'*) ∈ *C' *that satisfy *p *<*p'*. An edge from *C to C'* means that there exists at least one sequence where *C *occurs to the left of *C'*. The *weight (C,C') *of the edge (*C, C'*) is then defined as the number of sequences *i *with this property. For convenience purposes, we also add an initial vertex *v*_start _and a terminal one *v*_end_. The following result is quite easy to establish.

**Lemma 1**. *The set C is consistent if and only if SG(C) is a directed acyclic graph (DAG)*.

Finding a consistent set of partial columns amounts therefore to finding a set of partial columns whose succession graph is a DAG. To turn our possibly inconsistent set of subsets of sites $C=\{C_{1},C_{2},...,C_{p}\}$ into a consistent one, we proceed in two steps:

1. delete some edges of the succession graph *G *= *SG*(C) to turn it into a DAG,

2. transform the subsets *C_i _*themselves so that the succession graph of this new set of partial columns is itself a DAG.

For our applications, we will take $C=C_{k}$ described in the previous section, but the procedure we introduce here would work starting with any set of disjoint non-ambiguous subsets of S.

#### Getting a Directed Acyclic Graph

An optimal solution to the first problem would suppress a subset of edges of total *minimal weight *that yields a DAG. However, this is an NP-hard problem known as the *minimal (weighted) feedback arc set *problem. As a heuristic substitute, we successively remove the lowest weighted edges from the graph until all cycles have disappeared. Namely, let for *k *∈ ℕ the edge subset

$$E_{k}=\{(u,v)\in E|w(u,v)>k\;\;\text{or}\;\;u=v_{\text{start}}\;\text{or}\;v=v_{\text{end}}\},$$

and k* = min{*k *> 0|(*V*, *E_k_*) is a DAG}.

#### Removing Inconsistencies

**Figure 6:**
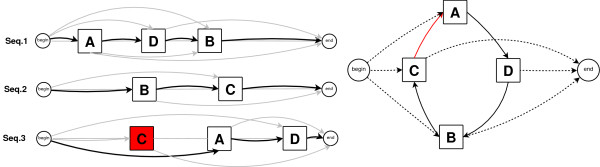
**Toy example for the removal of inconsistencies**. Removal of inconsistencies from a set of partial columns. On the left, the sites belonging to column "A" are denoted by a square box bearing that letter. On the right, the graph captures the immediate succession of the partial columns. The overall inconsistency can be detected by the cycle A→D→B→C→A. Assume that the DAG is obtained by removing the red edge C→A. The algorithm in section Getting an Acyclic Graph proceeds as indicated on the right. Namely, the longest path for each sequence is indicated with the bold black edges and the remaining edges from relation *R *are in grey. The result is the removal from the partial column "C" of the site indicated in red.

We describe now a method that will remove sites from the subsets *C_i _*in the set Csuch that the resulting set of partial columns *C' *is consistent. The algorithm tries to make the partial ordering on the partial columns induced by the DAG compatible with the linear partial ordering on the sites in the sequences, by removing a *minimal *set of positions from the partial columns. The subtle point is that deleting the edge (*u*, *v*) cannot be always interpreted directly as the removal of some positions that belong to partial columns *u *or *v*. The procedure is illustrated in Figure 6.

The acyclic graph $\left(V,E_{k^{*}}\right)$ can turn out to be disconnected, so we reconnect it by adding all the necessary edges of the form (*v*_start_, *u*) or (*u*, *v*_end_), and denote with G* the corresponding graph. Let ≤* be the partial order defined on *C *by the DAG G*. For each sequence *s*, let $C_{s}$ be the set of partial columns *C *of Chaving a (necessarily unique by definition) site (*s*, *j_C _*) in *s*. There are two order relations on Vs = $C_{s}$ U { *v*_start_,*v*_end_}, namely

• the total order ≼*_s _*induced by the natural order ≼ of S defined in section Methods,

• the partial order $\leq_{s}^{*}$ induced by the order ≤*defined by G*

The relation $R={≼}_{s}\cap \leq_{s}^{*}$ is the largest order which is a sub-relation of both ≼*_s _*and $\leq_{s}^{*}$ The total sub-orders, or *chains*, of the relation *R *are those subsets of occurrences of partial columns that are consistent. To minimise the number of lost sites, we choose a maximal chain.

More explicitly, let *G*^+ ^= (*V, E*^+^) be the transitive closure *TC*(G*) of G*. The graph *G*^+ ^is also a DAG and defines the same partial order on the set of partial columns. The graph *G_s _*= (*V_s_*, *E_s_*) of the relation *R *is defined on the vertex set *V_s _*by

$$(u,v)\in E_{s}\Leftrightarrow u,\;v\in V_{s},\;(u,v)\in E^{+}\;\text{and}\;u\;{≼}_{s}v.$$

Chains of *R *correspond to paths in *G_s_*. Let g*_s _*= (*v*_start_,*u*_1_,...,*u_n_*, *v*_end_) be a path from *v*_start _to *v*_end _in *G*_*s *_of maximal length. For all partial columns $C\in C_{s}$ such that C ∉ g*_s_*, remove the site (*s*, *j_C_*) from *C*. Let $C^{\circ }$ be the set of partial columns obtained after applying this procedure for all sequences *s *∈ *S*. The order in which they have been selected does not matter. If we wish to stress the difference between consistent and non-consistent partial columns, we will sometimes refer to the latter as *raw partial columns*.

**Lemma 2**. *The succession graph SG($C^{\circ }$) of the resulting partial column set is a DAG*.

*Proof*. Every direct transition between occurrences of partial columns in $C^{\circ }$ is encoded as an edge appearing in some longest path g in some graph *G_s_*. Therefore, every edge of the succession graph $G^{\circ }=SG(C^{\circ })$ corresponds to a *path *in the graph *G*^+^. Since *G*^+ ^is a DAG, the graph *G*°cannot have any cycle.

All current implementations of anchoring options take as input a list of pairs of matching positions. To obtain a set of anchor points from a set *C *of partial alignment columns, we consider all maximal segments of consecutive pairs of sites (*i, p*),...,(*i, p+k*) and (*i'*, *p'*),..., (*i'*, *p'+k*) such that every pair of sites (*i, p+l*) and (*i'*, *p'+l*),1 ≤ *l *≤ *k*, belongs to some partial alignment column $C_{j}\in C$.

## Results and Discussion

In order to evaluate the effect of introducing the MS4 partial columns in multiple alignments, we have used the reference protein multiple alignment benchmark database BAliBASE (release 3) [21]. As is usually done, we have only considered the core regions to assess the effect of the introduction of the partial columns in the MSA software. In order to do this, we have slightly waylaid DbClustal from its usual function, by including our MS4-based partial columns as anchors points encoded in BALLAST files, as explained in [7]. We have also used the anchoring option of DIALIGN 2 and included the partial columns as library les in T-Coffee.

For each of the reference sets in BAliBASE 3, we have examined and analyzed the performances of the aligners that accept anchors before and after the inclusion of two types of position subsets: (1) raw MS4 partial columns, computed according to section MS4 method (2) consistent MS4 partial columns, as obtained after applying the algorithm described in section Consistent Partial Columns.

The partial columns must be split into segments of pairwise matching positions, and attributed a weight. For a pair of segments of length *l *we set the weight to 10|*l*| for ClustalW and Dialign, and a uniform value of 100*M *for T-Coffee, where *M *is the number of sequences in the dataset. For each of the obtained alignments, we have computed the sum-of-pairs (SP) and total-column (TC) scores, and compared it to the scores obtained by the aligner alone. On DIALIGN, the results proved disappointing. With T-Coffee, no improvement nor degradation whatsoever was observed in the overwhelming majority of cases: there is a variation on less than 25 datasets over the whole BAliBASE3 (which consisting of 218), and a substantial one on about 5 only. These results are after all not so surprising, since both DIALIGN and T-Coffee already rely on local strategies. We will henceforth focus our discussion on the results obtained with ClustalW 2.0 alone. We omit "MS4" in what follows.

**Figure 7:**
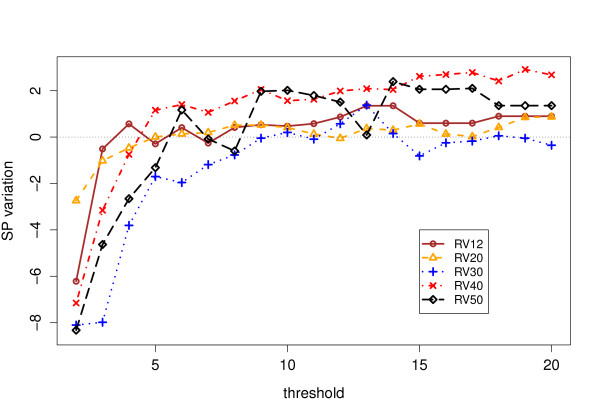
**SP Results for raw partial columns**. Sum-of-Pairs (SP) score improvements for ClustalW on BAliBASE 3 with raw partial columns. The plot shows the mean score variation Score(with anchors)-Score(without anchors) over all datasets for each reference set of the BAliBASE 3 benchmark (except RV11), against the minimum number *s*_min _of sequences where a partial column must be found to be included. The level 0 corresponds to a neutral effect.

**Figure 8:**
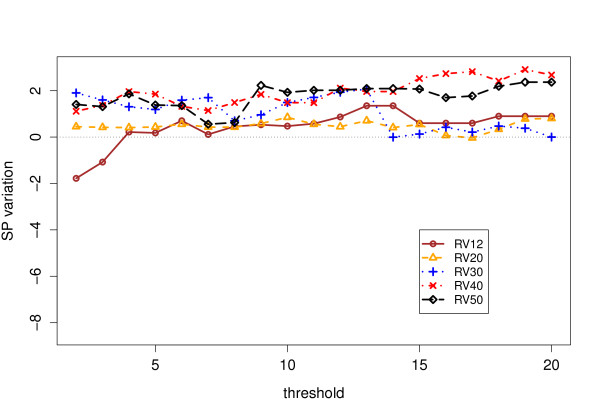
**SP Results for consistent partial columns**. Sum-of-Pairs (SP) score improvements for ClustalW on BAliBASE 3 with consistent partial columns.

Tables 1 and 2 contain the scores obtained by ClustalW 2.0 with and without our anchors, as well as those obtained by more modern aligners. We have reported the scores obtained for values of *s*_min _= 2, 6 and 12, which are somehow representative of the general trend, that we sum up as graphs in Figure 7,8, 9 &10. The consistency algorithm improves in every case the performance of ClustalW, while the partial columns computed by MS4 only improve it for *s*_min _= 6 and 12. Although the score improvements of our anchors on ClustalW are substantial, they remain inferior to those obtained by modern aligners.

**Table 1:** Sum-of-pairs score of ClustalW with anchor points on Balibase 3, compared to other usual aligners

Alignment	RV11	RV12	RV20	RV30	RV40	RV50
CLUSTALW 2.0	49.27	86.89	86.23	70.71	79.65	70.56

CW+pc. (2)	34.78	81.92	83.02	64.52	71.45	67.78

CW+pc. (6)	52.62	89.84	85.90	70.66	80.70	77.28

CW+pc. (12)	53.13	87.63	85.71	73.14	80.31	74.35

CW+c. pc. (2)	50.66	86.35	86.21	74.53	80.37	77.51

CW+c. pc. (6)	52.62	90.11	86.32	74.21	80.55	77.46

CW+c. pc. (12)	53.13	87.63	86.21	74.36	80.42	74.75

DIALIGN-TX	51.52	89.18	87.88	76.18	83.64	82.28

MAFFT 6.717b	66.19	93.36	92.72	87.08	92.19	90.25

MUSCLE 3.7	57.16	91.54	88.91	81.45	86.49	83.52

PROBCONS 1.12	66.97	94.12	91.68	84.53	90.34	89.41

T-COFFEE 7.81	66.77	94.08	91.62	83.81	89.96	89.43

This table gives the mean of the SP score on all data of each of the reference datasets of BAliBASE 3, for several multiple alignment schemes. CW+pc. (*k*) refers to the performance scores of ClustalW when supplied with the partial columns computed by MS4, for *s*_min _= *k*; while CW+c. pc. (*k*) refers to the consistent partial columns for the same value.

**Table 2:** Total-Column score of ClustalW with anchor points on Balibase 3, compared to other usual aligners

Alignment	RV11	RV12	RV20	RV30	RV40	RV50
CLUSTALW 2.0	24.00	72.32	20.44	26.87	40.04	34.21

CW+pc. (2)	13.48	62.48	16.15	19.41	29.21	27.14

CW+pc. (6)	21.34	74.95	24.18	27.01	39.07	41.67

CW+pc. (12)	17.33	66.36	24.96	28.69	33.01	31.71

CW+c. pc. (2)	25.89	70.42	26.18	30.24	37.28	40.06

CW+c. pc. (6)	21.34	75.59	26.18	29.65	37.34	38.56

CW+c. pc. (12)	17.33	66.36	25.25	27.27	32.92	31.71

DIALIGN-TX	26.81	75.69	30.78	38.90	45.17	47.05

MAFFT 6.717b	44.13	83.83	45.46	58.90	60.56	59.52

MUSCLE 3.7	32.06	80.90	35.30	41.19	45.32	46.39

PROBCONS 1.12	41.96	86.05	41.15	54.73	53.61	57.89

T-COFFEE 7.81	42.65	85.71	39.21	49.99	56.30	59.11

Same comments as in Table 1.

**Figure 9:**
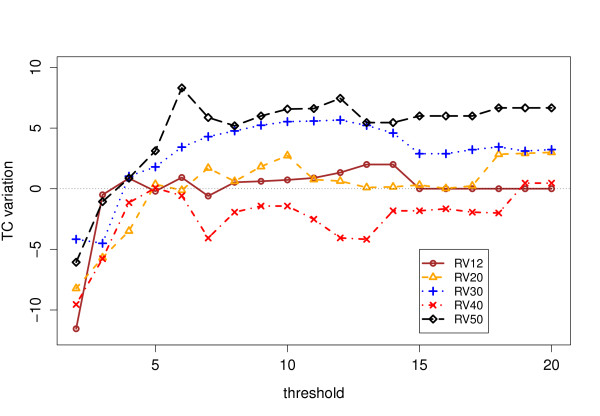
**TC Results for raw partial columns**. Total Column (TC) score improvements for ClustalW on BAliBASE 3 with raw partial columns.

**Figure 10:**
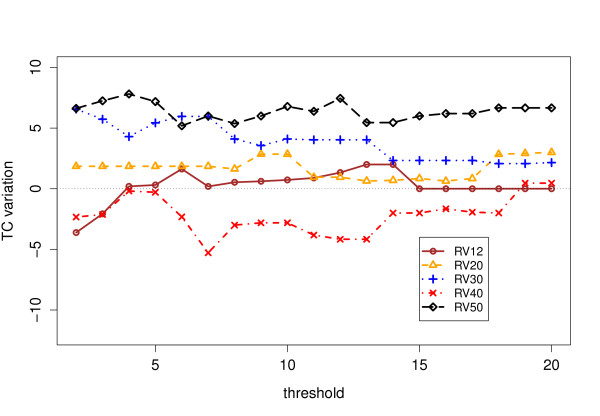
**TC Results for consistent partial columns**. Total Column (TC) score improvements for ClustalW on BAliBASE 3 with consistent partial columns.

Detecting a larger amount of correct similarities does not necessarily mean that the obtained alignment is better. Indeed, this effect could be obtained at the cost of including also a lot of wrongly aligned positions. To study this issue, we used the multiple alignment comparison tool aln_compare[4] by swapping arguments: usually the call aln_compare ref_al test_al computes among all pairs of aligned residues of the *reference alignment *ref_al, the proportion of residues which are present in the tested alignment test_al. If the arguments are swapped, the result returned counts the proportion of correct pairs among the core pairs aligned in the test alignment. A similar analysis is valid for the Total-Column score. These measures can be considered as *specificity scores*. We have reported in Table 3 the specificity scores (for both measures of specificity-SP and TC), obtained by the ClustalW 2.0 alignments alone, and for ClustalW with our consistent partial columns for *s*_min _= 6, which appears from Tables 1 and 2 as being the best overall combination.

**Table 3:** Specificity scores for ClustalW with anchor points on Balibase 3

Alignment	RV11	RV12	RV20	RV30	RV40	RV50
CLUSTALW 2.0	71.50/28.97	93.73/76.67	94.87/21.26	87.39/29.84	93.18/41.99	87.83/37.59

CW+c. pc. (6)	75.50/27.18	95.60/79.56	95.73/26.97	89.23/33.12	92.76/39.18	91.96/41.17

The specificity scores obtained by Clustalw 2.0 for SP and TC evaluation schemes, as compared with the scores obtained with the consistent partial columns for *s*_min _= 6.

### SP scores

We can observe from Figure 7 that, as a general trend, the inclusion of raw partial columns induces a general degradation of performance for *s*_min _< 5, and a global improvement above this threshold (except for RV30). The score degradation for *s*_min _< 5 shows that the raw partial columns include inconsistent similarities for these values, which are eliminated by requiring that a column span a minimum number of sequences in order to be considered.

Figure 8 illustrates the effect on the SP scores of introducing consistent partial columns. A perceptible improvement (from 0.5 to 2 points) is then observed, and no degradation of the SP scores for weak values of *s*_min _(except for RV12) is to be seen. This indicates that the consistency algorithm manages to suppress inconsistent similarities even when they only concern as few as 2 sequences. More generally, the improvement due to the inclusion of the consistent partial columns is clearer for RV40 and RV50.

This result fits with the expectations, since these two datasets contain respectively large (C- or N-) extensions and large internal deletions. It is well known that supplying local information help global aligners to deal with large indels.

### TC scores

Figures 9 and 10 show the variation of TC score with respectively raw and consistent columns. The TC score is much more stringent, since a single mistake in a column as compared to the reference alignment results in a score of 0 for the considered column. As for the SP score, the degradation that can be observed for *s*_min _< 5 with raw partial columns disappears as soon as the columns have been filtered by the consistency algorithm, and gives on the contrary a perceptible improvement (with the notable exception of RV40). This means that, although the actual number of correctly aligned *pairs *does not greatly increase (see Figure 8), the improvement concerns essential columns of the core reference alignment. If the consistent partial columns are able to improve the TC scores, it shows that they can find previously undetected local similarities for a subset of sequences where the similarity was missed and now can be included for all sequences, because the TC score will only raise if a column is aligned correctly in all sequences. The improvement is more perceptible for RV30 and RV50. The dataset RV30 contains highly divergent sequences and RV50 large indels, as we recalled before.

## Discussion

**Figure 11:**
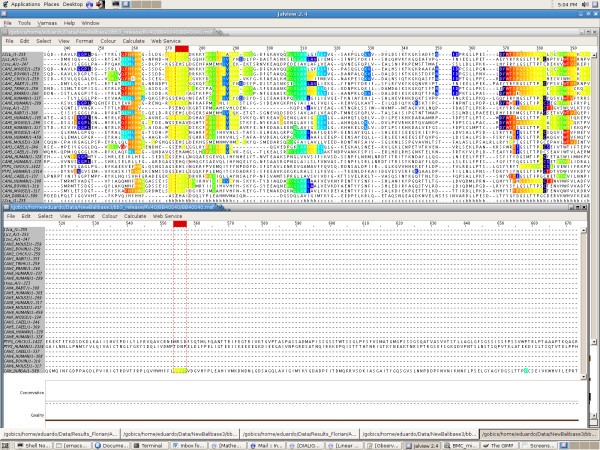
**Screenshot of Jalview visualization of alignment on dataset BB40040**. Jalview screenshots of two different regions of the reference multiple alignment of dataset BB40040, featuring our consistent partial columns for *s*_min _= 2 shown in colour. The local similarities detected by NLD that are responsible for the 0 TC score are shown as the yellow-green coloured sites enclosed in the red dotted region. This is a (rare) example where a local similarity involving up to 18 sequences turns out to be biologically wrong. The offset on the columns resulting from the wrong alignment of the last sequence is sufficient to induce at least one mistake per column along a large part of the core region.

It is somehow surprising that we do not get an improvement on the TC score for RV40, since this is the dataset that is used to test large C- and N-terminal insertions. For this set, it often happens that ClustalW does not align correctly any single core column, whereas supplied with our consistent partial columns, it will manage to correct this behaviour, resulting in a great improvement in score. However, the weak mean performance of the partial column anchoring on the TC score for RV40 is essentially due to 2 alignments out of the 49 composing the dataset. For BB40044, ClustalW aligns correctly 84% of the columns, whereas for *s*_min _≤ 13 our anchors introduce mistakes, resulting in a TC 0 score. The same happens with BB40040 for which ClustalW finds 70% of correct columns, and none with our consistent partial columns (except for *s*_min _≥ 19). A closer examination of the alignments shows that, for BB40040, MS4 detects (rightly) a similar region that extends over 18 sequences, but it turns out that for 1 sequence it should not be aligned with the 17 others. The resulting offset of the positions in this last sequence runs whole columns along the core region (see Figure 11). This unexpected phenomenon however only slightly affects the number of aligned pairs, which is consistent with the fact that otherwise the SP score is in the average of the improvements observed on the other reference sets.

**Figure 12:**
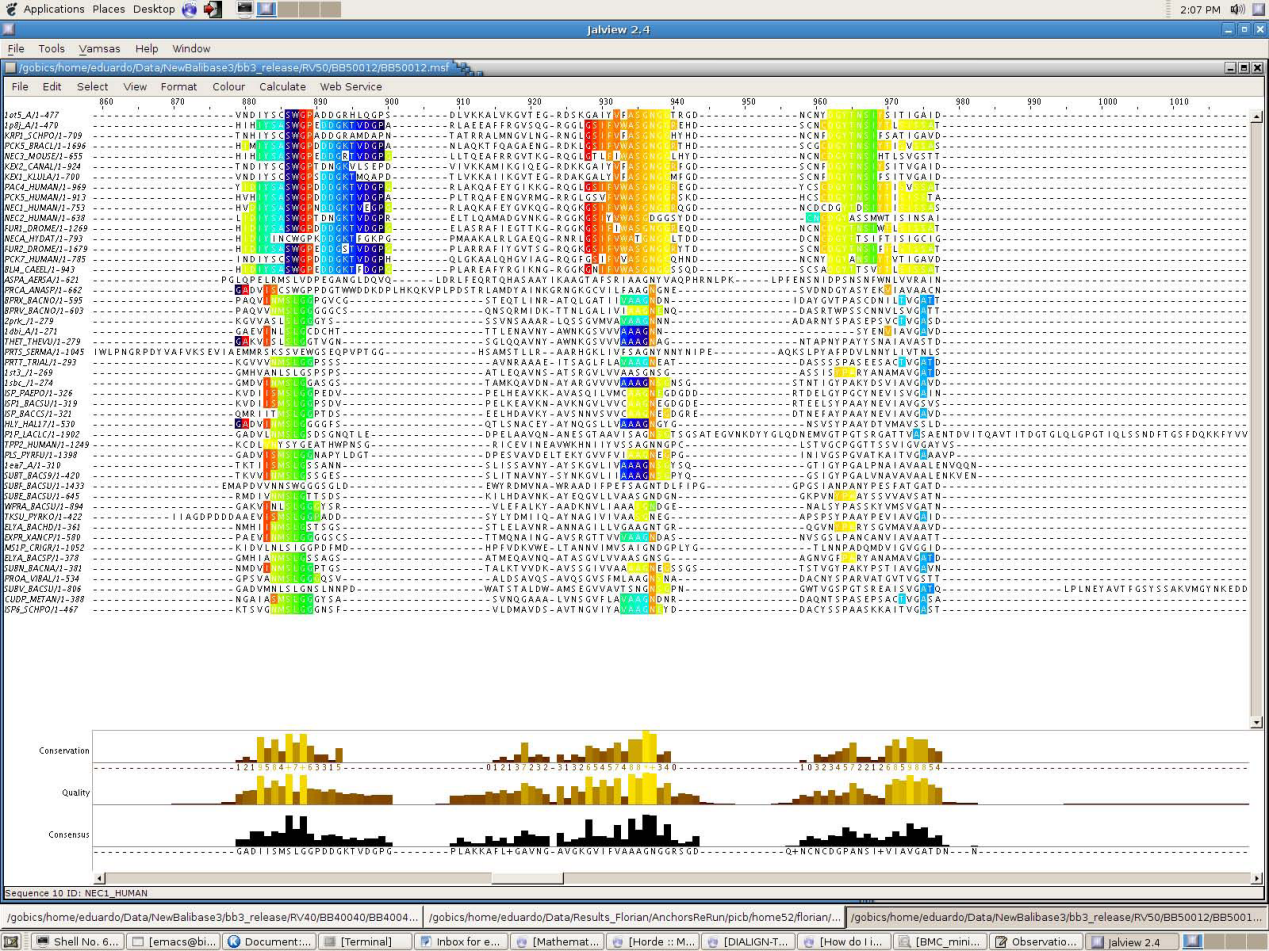
**Screenshot of Jalview visualization of reference alignment for dataset BB50012**. Jalview screenshot of the consistent partial columns for *s*_min _= 3 among one of the core regions of the BAliBASE reference alignment for dataset BB50012. The partial columns (shown in colour) correspond to parts of correct columns in a sufficient amount to correct the overall behaviour of ClustalW, resulting in a TC score of 44 (instead of 0). Notice the clear separation of the partial columns between eukaryotes and prokaryotes.

It is to be noted that on RV50, on the contrary, the inclusion of consistent partial columns always result in an improvement of the TC score, whatever the considered dataset. Here in Figure 12, we show the consistent partial columns on the reference set BB50012, where the TC score jumps from 0 to 44. Notice that the columns appear to be split in two groups, which turn out to correspond to the separation between eukaryotes and prokaryotes. This feature illustrates why the MS4 can be used as an efficient alignment-free classification tool [19].

Finally, the specificity scores reported in Table 3 seem to indicate that the sensitivity score improvements are indeed a result of a larger number of detected similarities that are relevant. Note that the unsatisfactory behaviour of TC on RV40 reflects also on the specificity score. In Table 4, we have reported the times taken on average by the two steps of our anchor selection algorithm. The consistency step has to be repeated for each sequence: this accounts for the higher figures for RV20 and RV30, which consist of more sequences on average than the other datasets. The program used is still a prototype, and has not been optimised for performance; nevertheless, the time required remain reasonable and does not seem to be an obstacle to using this feature on datasets of gene-sized alignable sequences.

**Table 4:** Time performances for the steps of the consistency algorithm

Algorithm step	RV11	RV12	RV20	RV30	RV40	RV50
MS4	0.83	1.61	18.83	34.08	10.91	13.25

Consistency	0.73	5.22	134.10	160.07	51.76	65.14

Mean values of the time (in s.) taken by the MS4 step and the consistency step for the anchor computation on BAliBASE 3.

We have also used both types of partial columns with DIALIGN. However, as mentioned, probably since this aligner is already based on local similarities, we didn't observe any improvements on BAliBASE 3. Further investigations seem to show that MS4 is here to blame. When partial alignment columns are constructed from the pairwise similarities computed by DIALIGN, we have shown in [22] that the consistency algorithm successfully removes inconsistencies, resulting this time in an improvement of performance with DIALIGN 2. This supports the idea that a more refined criterion for selecting the nodes in the partition tree than the one currently implemented in the MS4 method is required to be successfully applied as a local similarity detector that performs well on more modern aligners. At any rate, MS4 seems more adapted to alignment-free classification. According to our experience, the partial columns obtained by MS4 are nevertheless useful for the visual expertise of alignments, for they highlight local homologies (for instance when used with a multiple alignment editor like Jalview), which are easier to visualise than the usual simple substitutions schemes used by these editors.

## Conclusions

The introduction of our MS4-based partial columns give therefore encouraging results. The overall influence of their inclusion can be summed up in two principal observations. The introduction of local information results in an improvement of the correctness of ClustalW, as already observed by the authors themselves, who developed DbClustal for this goal. Initially, DbClustal uses local fragments based on BLAST searches (local similarities with sequences stored in generalist protein databanks). The inclusion of user-defined anchor points being also possible, we have in this way been able to assess the improvement of performance that results from the inclusion of these local primary sequence-based similarities, constructed without score matrices or sliding window of predefined length. With the local aligners for which the inclusion of anchor points is possible, the results are not conclusive, especially with DIALIGN, although they happen to have quite a neutral effect on T-Coffee. It is unfortunate that the anchoring option is not featured in any other aligner, especially any other global aligner, to be able to give more insight on the usefulness of the construction presented here.

The improvement obtained for ClustalW is most perceptible for datasets containing sequences of unequal lengths, and the computation of MS4 partial columns seems then justified in view of the gain in accuracy they provide. In other respects, the computation of consistent partial columns can help the eye-expertise of multiple alignments, for the number of obtained position subsets is quite reasonable, and, as the TC score performance seems to indicate, their visualization allows to correct whole columns in the alignment, since they appear to correspond to conserved zones in the considered sequences (like in Figure 12 for instance). We have moreover introduced an algorithmic approach that can be further explored. The consistency algorithm can be used with other local similarities as input, as already tested with success on DIALIGN [22]. These results encourage us to improve our approach on several points. In particular, the mere filtering of edges of the succession graph by their weight to get a DAG in section Getting a Directed Acyclic Graph is overly simplistic (although effective). We are currently exploring more refined ways of getting a DAG, in order to reduce the number of erased edges. Another interesting feature would consist in splitting the contradicting partial columns into subsets of similarly behaved sites. These algorithmic improvements could then fit in a general tool for making local similarities consistent.

## Authors' contributions

EC and FP have developed the methods and conducted the tests, FP and CD have performed the expertise, and all three authors have drafted, read and approved the manuscript.
